# ZLM-7 inhibits the occurrence and angiogenesis of breast cancer through miR-212-3p/Sp1/VEGFA signal axis

**DOI:** 10.1186/s10020-020-00239-2

**Published:** 2020-11-13

**Authors:** Xuan Li, Zi-Zheng Zou, Min Wen, Yuan-Zhu Xie, Kun-Jian Peng, Tiao Luo, Su-You Liu, Qin Gu, Ji-Jia Li, Zhi-Yong Luo

**Affiliations:** 1 Molecular Biology Research Centre, Hunan Province Key Laboratory of Basic and Applied Hematology, Hunan Key Laboratory of Animal Models for Human Diseases, School of Life Sciences, Central South University, Changsha, 410008 Hunan China; 2 Xiangya Hospital, Central South University, Changsha, 410008 Hunan China; 3 School of Pharmaceutical Sciences, Central South University, Changsha, 410013 China; 4 Hunan Key Laboratory of Oral Health Research, Xiangya Stomatological Hospital, Xiangya School of Stomatology, Central South University, Changsha, 410008 Hunan China

**Keywords:** Breast cancer, ZLM-7, miR-212-3p, Sp1, VEGFA

## Abstract

**Background:**

Breast cancer (BC) is a common malignant tumor with poor prognosis. Angiogenesis is related to the growth and progression of solid tumors and associated with prognosis. ZLM-7, SP1, VEGFA and miR-212-3p were associated with BC angiogenesis and proliferation, however the detailed mechanism was not clear. This study aimed to reveal the regulatory mechanism of angiogenesis of BC.

**Methods:**

BC cell lines were treated with 10 nM ZLM-7 for 8 h. We detected protein expression level by western blot and RNA expression level by qRT-PCR. Overexpression or inhibition of miR-212-3p is performed using miR-212-3p mimics or miR-212-3p inhibitor, Sp1 overexpression using pcDNA3.1 vector. Angiogenesis was analyzed by co-culturing BC cell lines and HUVEC cells. To evaluate regulatory relationship between miR-212-3p and Sp1, dual luciferase assay was performed. Besides, the direct interaction between Sp1 and VEGFA was analyzed by ChIP. Migration and invasion were analyzed by transwell assay and proliferation was detected by clone formation assay. In mice xenograft model developed using BC cells, we also detected angiogenesis marker CD31 through immunohistochemistry.

**Results:**

ZLM-7 up-regulated miR-212-3p and inhibited invasion, migration, proliferation and angiogenesis of BC, while miR-212-3p inhibitor antagonized such effects. Binding sequence was revealed between miR-212-3p and Sp1, and expression of Sp1 was inhibited by miR-212-3p on both protein and mRNA level. Sp1 could interact with VEGFA and promoted its expression. Overexpression of miR-212-3p inhibited migration, invasion, proliferation and angiogenesis of BC cell lines, while Sp1 overexpression showed the opposite effect and could antagonize these effects of miR-212-3p overexpression. ZLM-7 decreased VEGFA expression, which was rescued by co-transfection with miR-212-3p inhibitor. Similar, ZLM-7 could inhibit tumor growth and angiogenesis through the miR-212-3p/Sp1/VEGFA axis in vivo.

**Conclusions:**

ZLM-7 could directly up-regulate miR-212-3p in BC. MiR-212-3p could inhibit VEGFA expression through Sp1, thereby inhibiting angiogenesis and progression of BC.

## Background

Breast cancer (BC) is a common malignant tumor, originated from the breast epithelial tissue of women, which is considered to be one of the top ten common cancers in the world (Forouzanfar et al. 2011). According to cancer statistics 2020, over 1.7 million cases were newly diagnosed per year. Among these patients, more than 30% would eventually suffer metastasis and their 5-year-survival rate was less than 25% (Siegel et al. 2020). Today, the conventional and effective therapies for BC include targeted therapy, immune therapy, surgical resection and chemotherapy. Despite significant improvements in diagnosis and treatment, the prognosis has been poor due to delayed diagnosis, high incidence of recurrence and metastasis, and adverse effects of drugs (Bonotto et al. 2014; Brok et al. 2017). Therefore, investigating on mechanisms of BC development, recurrence and metastasis is crucial for a better understanding and development of new therapeutic options.

Angiogenesis is the product of new blood vessels growing from the original blood vessels, which is related to the growth and progress of solid tumors (Ramjiawan et al. 2017). If there is no angiogenesis, the size of most solid tumors cannot exceed 1–2 mm^3^, because the tissue oxygen diffusion limit is 100–200 μm (Viallard and Larrivee 2017). Besides, angiogenesis would also promote metastasis of BC. The risk of metastasis would increase 1.59 fold if there were 10 micro vessel increase in a 200 × field under microscope (Weidner et al. 1991). Angiogenesis involves several steps, including degradation of surrounding extracellular matrix, migration of endothelial cells, proliferation of endothelial cells and transformation of endothelial cells into tubular structures (Ronca et al. 2017). The microtubule targeting inhibitor (MTA) has the effects of destroying blood vessels and anti-angiogenesis. Combretastatin A-4 (CA-4) is one of the most effective MTAS. It has a strong affinity for microtubule proteins, leading to the instability of cytoskeleton (Ma et al. 2016). ZLM-7 is a *cis* restricted sulfide derivative of CA-4, which has the same antitumor activity as CA-4, but its toxicity is lower than CA-4 (Su et al. 2016). Previous studies had shown that ZLM-7 treatment could reduce the proliferation and angiogenesis of BC cells (Su et al. 2016).

MiRNA is a kind of endogenous small RNA with 20–24 nucleotides in length, which has many important regulatory functions in cells. Each miRNA can have multiple target genes, and several miRNAs can also regulate the same gene and fine regulate the expression of this target gene (Afonso-Grunz and Müller 2015; O'Carroll and Schaefer 2012). MiRNA have was associated with multiple stages during regulation of angiogenesis (Tiwari et al. 2018). For example, miR-29b could inhibit angiogenesis and tumorigenesis via targeting AKT3 and inhibiting VEGF and c-myc (Li et al. 2017a), miR-1908, miR-199a could synergistically inhibit ApoE/LRP1/LRP8 signal pathway and suppress angiogenesis and metastasis in melanoma (Pencheva et al. 2012). MiR-212-3p is another miRNA involved in angiogenesis regulation in various types of cancers. It was reported that miR-212-3p was significantly down-regulated in human bladder cancer tissues and its over expression could inhibit the proliferation of bladder cancer cells (Wu et al. 2019). It could also inhibit angiogenesis and proliferation of glioblastoma by targeting SGK3 (Liu et al. 2015). Through our previous transcriptome sequencing, we found that ZLM-7 treatment could significantly up-regulate the expression of miR-212-3p in BC tissues.

Specific protein 1 (Sp1) is one of the earliest identified transcription factors (Beishline and Azizkhan-Clifford 2015). The abnormal activation of Sp1 could up-regulate the expression of tumor related factors and angiogenic factors, thus providing a good microenvironment for tumor growth, and promoting the proliferation, metastasis and angiogenesis of colon, gastric and pancreatic tumors and regulate apoptosis (Beishline and Azizkhan-Clifford 2015; Liu et al. 2018; Torabi et al. 2018). Sp1 could interact with the promoter of vascular endothelial growth factor A (VEGFA), and up-regulate its expression, which promotes the proliferation of vascular endothelium, angiogenesis and vascular permeability, thus promoting the growth and metastasis of tumor via binding with VEGF receptor 2 (VEGFR2) (Chen et al. 2018; Vizcaino et al. 2015). However, the specific mechanism of Sp1 in angiogenesis regulation in BC is not clear.

It was previously reported that Sp1 could directly promote the expression of VEGFA, and that miR-212-3p could inhibit angiogenesis and proliferation of glioblastoma (Liu et al. 2015; Chen et al. 2018), and ZLM-7 could increase the expression of miR-212-3p in BC cell lines. We thus hypothesized a regulation network between Sp1, miR-212-3p and ZLM-7 in BC. The purpose of this study is to examine the molecular regulatory mechanism of ZLM-7 on miR-212-3p/Sp1/VEGFA signal axis in BC. Therefore, we conducted the following experiments to explore their biological functions and its underlying mechanism in BC, we hope to provide new insights for BC prediction and treatment.

## Methods

### Cell culture and treatment

MCF-7 and JIMT-1 were obtained from ATCC. HUVECs and 293T were purchased from the Shanghai Institute for Biological Sciences, Chinese Academy of Sciences. MCF-7 was cultured in MEM (Gibco, NY, USA) + 0.01 mg/ml insulin + 10% FBS (Gibco, NY, USA) + 100 U/ml P/S (penicillin + streptomycin) (Gibco, NY, USA). JIMT-1 was cultured in H-DMEM (Gibco, NY, USA) + 10% FBS + 100 U/ml P/S. HUVECs and 293T were cultured in DMEM + 10% FBS + 100 U/ml P/S. Cells were tested without contamination with mycoplasma. Cells were cultured in a humidified incubator at 37 ℃ with 5% CO_2_. Cells were passaged at 90% confluency and seeded at 25% confluency unless otherwise stated. For ZLM-7 treatment, BC cell lines were treated with 10 nM ZLM-7 for 8 h. Proliferation, migration, invasion and tube formation assays were performed after ZLM-7 treatment.

### Transfection

MiR-212-3p mimics, mimics negative control (NC), miR-212-3p inhibitor and inhibitor NC were purchased from GenePharma (Shanghai, China). Sp1 (+) or pcDNA3.1 were designed and purchased from GenePharma (Shanghai, China). Briefly, BC cells were seeded at 25% confluency, cultured in medium under 5% CO_2_ condition at 37 ℃ for 24 h cells and transfected at 50% confluency with lipofectimin2000 (Thermo Fisher Scientific, MA, USA) following manufactures’ instructions. Cells were harvested at 48 h for further experiments.

### Transwell assay for migration and invasion

Place the transwell chamber in 24 well plates to form a two-compartment system. Cells were collected by centrifuge at 3000 rpm, 4 ℃ for 5 min. Cells were then resuspended, washed twice and then resuspended in serum-free medium at a density of 10^6^ cells/ml.

For migration, 100 μl of cell suspension was added to the upper compartment and 0.8 ml medium containing 5% FBS was put in the lower compartment. After 24 h of regular culture, the porous membrane was isolated, fixed with methyl alcohol, and stained with crystal violet. Cells found in at least 20% of the area of the filter was counted under immunofluorescence microscope (Leica, Allendale, NJ, USA).

For invasion, thaw Matrigel on ice and dilute Matrigel with serum free medium by 1:8, and add 40 μl dilutions in the upper compartment (all experiments were conveyed on ice). Then, 100 μl of cell suspension was added to the upper compartment and 0.8 ml medium containing 5% FBS was put in the lower compartment. After 24 h of regular culture, the porous membrane was isolated, fixed with methyl alcohol, and stained with crystal violet. Cells found in at least 20% of the area of the filter was counted under immunofluorescence microscope (Leica, Allendale, NJ, USA).

### Clone formation assay

Cells were collected by centrifuge at 1500 rpm, 3 min. Pellets were re-suspended by basic medium. Adjust cell density of the mixture to 2000 cells/ml and seed 100 μl of the mixture into each well of 6 well plates. During culturing, observe cell status every 2 days, until most wells reached at least 50 clones. Cells were then fixed by 4% PFA and stained by GIEMSA. Pictures were observed and photographed under a microscope (Plympus CKX41).

### Angiogenesis and tube formation

After MCF-7 or JIMT-1 cells was treated and transfected, then, cultured for 24 h. Their media were collected as so called ‘conditioned medium’. To detect angiogenesis, HUVEC were treated with these conditioned medium for 24 h. Angiogenesis assay was performed by in vitro Angiogenesis Assay Kit (ab204726; Abcam, Shanghai, China) following manufactures’ instructions. Add 50 μl of thawed Extracellular Matrix Solution to each well of a pre-chilled (on ice) white 96-well sterile cell culture plate. Incubate for 1 h at 37 ℃ to allow the solution to form a gel. Use 2 × 10^4^ endothelial cells/well for a white 96-well plate using 100 μl media/well. Add cells onto the solidified Extracellular Matrix gel or control wells. Add angiogenesis factors/regulators to the desired wells. Incubate cells for 18 h in a 37 ℃ incubator containing 5% CO_2_. Carefully remove the medium and wash the wells with 100 μl of Wash Buffer to remove serum. Add 100 μl of Staining Dye Working Solution to each well. Incubate for 30 min at 37 ℃. Examine the endothelial tube formation using light and fluorescence microscopy (green filter) (DMI6000B; Leica, Allendale, NJ, USA).

### Bioinformatics analysis and dual-luciferase assay

Bioinformatics analysis using starBase (https://starbase.sysu.edu.cn) was used to identify potential binding targets of miR-212-3p and to locate the binding sequences within the 3′ untranslated region (3′-UTR) of Sp1. The wild-type (WT) and mutant (MUT) Sp1 sequences were synthesized and inserted into the pmirGLO vector systems (Promega, Wisconsin, USA), respectively. Point mutations of the miR-212-3p targeting sites in the Sp1 3′-UTR were directly synthesized using the QuickChange Multiple Site-directed Mutagenesis Kit (Stratagene, La Jolla, CA, USA). 293T Cells were seeded (4 × 10^4^ cells/well) in triplicate in 24 well plates. Luciferase reporter system was transfected into 293T cells as indicated. After 48 h of transfection, luciferase assay was conveyed using Dual Luciferase Reporter Assay System (Promega, Wisconsin, USA) according to the manufacturer’s instruction. Briefly, supernatants were removed. Cells were rinsed by PBS for 3 times and lysed with lysis buffer for 20 min at room temperature. After that, 100 μl supernatant were transferred into luminometer tubes, mixed with 20 μl luciferase assay reagent and signals were detected on a GloMax20/20 luminometer (Promega, Wisconsin, USA).

### Chromatin-immunoprecipitation (ChIP)

Briefly, cells were collected, lysed, mixed with 100 μl of antibody-conjugated dynabeads and incubated overnight at 4 ℃ on rotating platform. Collect beads with magnet concentrator and wash with RIPA buffer twice and with TE buffer once. Add 50 μl of elution buffer and vortex briefly to resuspend the beads. Incubate for 10 min at 65 ℃. Vortex briefly every 2 min during incubation. Centrifuge the tube for 30 s at maximum speed, and then transfer the liquid to a new tube. Add 120 μl of elution buffer to the supernatant in the new tube. Reverse the cross-links overnight in a 65 ℃ incubator. DNA was purified and then resuspend it in 30 μl of TE buffer containing 10 μg of RNase A. Incubate for 2 h at 37 ℃. Purify the DNA using a QIAGEN QIAquick PCR purification kit.

### Xenograft model analysis

Ethical approval for animal experiments was obtained from Central South University Animal experiments were carried out in strict accordance with the recommendations in the guide for the care and use of laboratory animals. All surgeries were performed under chloral hydrate anesthesia, and all efforts were made to minimize suffering. BALB/c nude mice (6 weeks old) were purchased from Shanghai Laboratory Animal Center (Shanghai, China). All mice were housed in a specific-pathogen-free facility with a 12 h light: dark cycle at a temperature of 21 ℃ ± 2 ℃ and a relative air humidity of 55% ± 10%. ZLM-7 was administrated 15 mg/kg/day by intraperitoneal injection based on previous reports (Su et al. 2016). MiR-212-3p inhibitor dissolved in Entranster-in vivo (Engreen Biosystem, China) was injected through caudal vein 1 mg/kg weekly (Zhang et al. 2019). Each mouse was subcutaneously inoculated with 5 × 10^6^ cells into the back next to the right front limb. Tumors were measured every 5 days with a standard caliper, and tumor volumes were calculated as follows: tumor volume (mm^3^) = [tumor length (mm) × tumor width (mm)^2^]/2. At the end of the experiment (4 weeks), the animals were anesthetized. Tumors were weighed after separated from the surrounding muscles and dermis.

### Immunohistochemistry (IHC)

Briefly, tumors sections were washed twice by TBST and blocked by 3% serum for 10 min. Sections were then incubated with the primary antibody in a moist chamber for 4 h at room temperature and wash sections by TBST three times. Sections were then incubated with biotin-conjugated secondary antibody and incubate for 45 min, and washed twice in TBST. Then incubate sections in 0.1% NaN_3_ and 0.3% H_2_O_2_ for 30 min. After washing sections with TBST for 3 times, add HRP-conjugated avidin and incubate for 20 min. Wash three times by TBST and add 50 ml of the developing solution in dark. Incubate until complete, dense staining is obtained, but background is still low. Wash thoroughly in tap water. Transfer to TBS 0.05 M pH7.5 + 0.01% Tween 20. CD31 (CST#77699) antibody was purchased from cell signaling technology (CST) and diluted 1:100.

### Quantitative real time polymerase chain reaction (qRT-PCR)

RNAs were extracted from BC cell lines and mice tumor tissues. We extracted RNA by TRIzol reagent (Invitrogen, CA, USA) and synthesized cDNA by PrimeScript RT reagent Kit (Takara #RR037A). Real time PCR was conveyed on ABI Prism 7500 system (Applied BioSystems, CA, USA) in a 96 well plate using SYBR Premix Ex Taq II (Takara RR820A). Reaction system was set as: 40 cycles of 94 ℃ for 5 s, 60 ℃ for 34 s, and 72 ℃ for 30 s. The relative expression level was calculated using 2^−∆∆Ct^ method. MiR-212-3p: forward 5′-CGC GAG ATC AGA AGG TGA TT-3′ and reverse 5′-GTC GTA TCC AGT GCA GGG TCC GAG GTA TTC GCA CTG GAT ACG ACA GCC AC-3′. Sp1: forward 5′-GAC AGG ACC CCC TTG AGC TT-3′ and reverse 5′-GGC ACC ACC ACC ATT ACC AT-3′. VEGFA: forward 5′-CTA GGA AAA TCG ACC AGA TGC C-3′ and reverse 5′-AGC GCG TGT TGC AGG TCT TGG AT-3′. U6: forward 5′-CTC GCT TCG GCA GCA CA-3′ and reverse 5′-AAC GCT TCA CGA ATT TGC GT -3′.GAPDH: forward 5′-CCA GGT GGT CTC CTC TGA-3′ and reverse 5′-GCT GTA GCC AAA TCG TTG T-3′. U6 or GAPDH was used as an internal control.

### Protein extraction and western blot

Total proteins were extracted from cultured cells or xenograft tumors using RIPA buffer (Thermo Fisher Scientific, Waltman, MA, USA) supplemented with protease inhibitor cocktail (Cell Signaling Technology, Danvers, MA, USA). Protein concentration was analyzed by BCA kit (ThermoFisher, MA, USA) following manufactures’ instructions. For each sample, 20 μg protein were loaded onto SDS-PAGE and separated. Proteins were transferred to nitrocellulose (NC) membrane and blocked with 5% BSA at room temperature for 1 h. Then, membranes were incubated with primary antibody at 4 ℃ overnight. After repeated washing by TBST buffer (20 mM Tris, 137 mM NaCl, 0.1% Tween-20, pH 8.0), membranes were incubated with secondary antibodies at room temperature for 1 h. Signal were detected by enhanced chemiluminescence (ECL) western blotting detection system. Antibodies of Sp1 (CST#5931), VEGFA (CST#8908), GAPDH (CST#5174) and MMP-2 (CST#40994) were purchased from cell signaling technology (CST) and diluted 1:1000 according to manufactures’ instructions.

### Statistical analysis

All data were analyzed using GraphPad Prism 7 (GraphPad Software, Inc., LA Jolla, CA, USA) and presented as the means ± SD. The results were analyzed using Student *t* test (two groups) or one‐way analysis of variance (ANOVA) (multiple groups) as appropriately. The *P* < 0.05 was considered statistically significant. All experiments were repeated three times.

## Results

### ZLM-7 inhibited the migration and invasion of BC cells by up regulating the expression of miR-212-3p

**Fig. 1:**
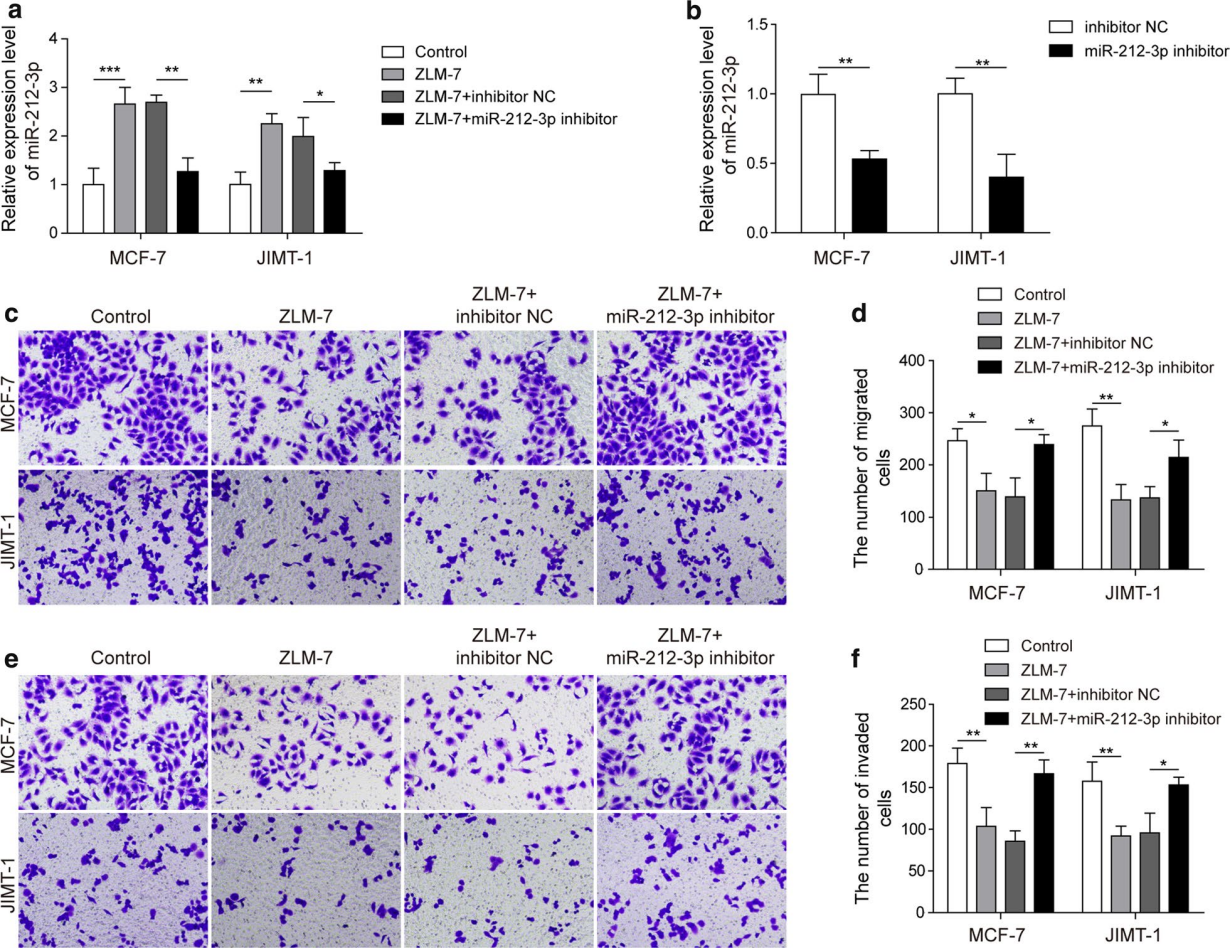
ZLM-7 inhibited the migration and invasion of BC cells by up regulating the expression of miR-212-3p. **a** Relative expression of miR-212-3p in MCF-7 and JIMT-1 treated with ZLM-7, miR-212-3p inhibitor or NC by qRT-PCR analysis. **b** Expression level of miR-212-3p in MCF-7 and JIMT-1 after transfection with miR-212-3p inhibitor. **c**, **d** Cell migration of MCF-7 and JIMT-1 detected by transwell assay after ZLM-7 and miR-212-3p inhibitor treatment. **e**, **f** Cell invasion of MCF-7 and JIMT-1 detected by transwell assay after ZLM-7 and miR-212-3p inhibitor treatment. *P < 0.05, **P < 0.01, ***P < 0.001

To evaluate whether ZLM-7 administration could affect the expression of miR-212-3p and inhibit the migration and invasion of BC cell line, we conveyed qRT-PCR analysis and transwell assay in two independent BC cell lines MCF-7 and JIMT-1. MiR-212-3p was up-regulated in both cell lines after ZLM-7 and ZLM-7 plus inhibitor NC treatment, but miR-212-3p inhibitor could reverse the promotion effect of ZLM-7 on miR-212-3p (Fig. 1a). The expression level of miR-212-3p was down-regulated when cells were transfected with miR-212-3p inhibitor (Fig. 1b). ZLM-7 treatment reduced the migration ability of BC cells, miR-212-3p inhibitor antagonized the inhibition effects of ZLM-7 (Fig. 1c, d). Similar results were observed in invasion ability. Invasion was inhibited by ZLM-7 administration, and rescued by miR-212-3p inhibitor (Fig. 1e, f). These results demonstrated that ZML-7 could inhibit migration and invasion of BC cells via upregulating miR-212-3p.

### ZLM-7 inhibited the proliferation and angiogenesis of BC cells by up regulating miR-212-3p

**Fig. 2:**
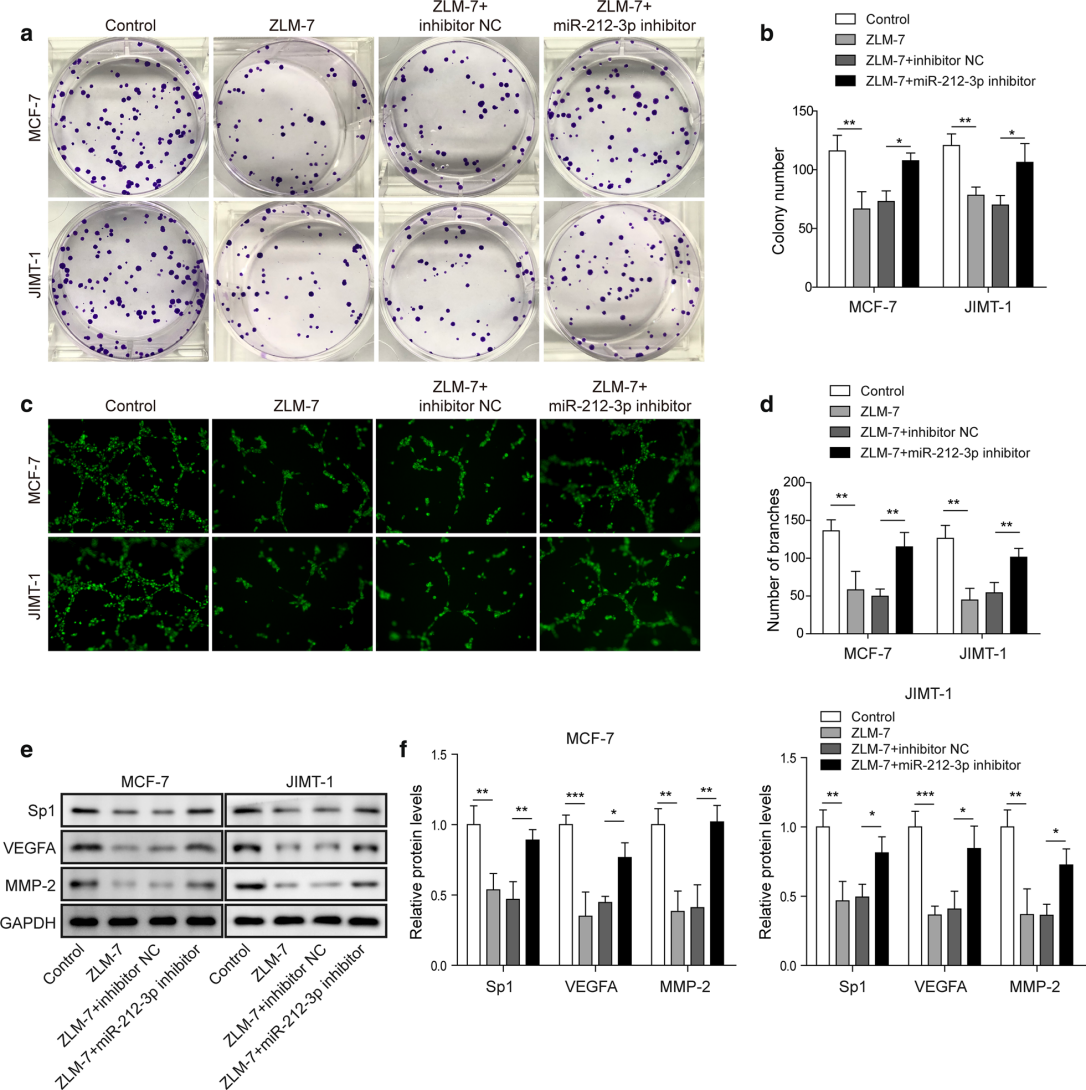
ZLM-7 inhibited the proliferation and angiogenesis of BC cells by up regulating the expression of miR-212-3p. **a**, **b** Cell proliferation of MCF-7 and JIMT-1 was determined by colony formation in cells treated with ZLM-7 and miR-212-3p inhibitor. **c**, **d** Tube formation analysis in MCF-7 and JIMT-1 treated with ZLM-7 and miR-212-3p inhibitor. **e**, **f** Western blot analysis in of MCF-7 and JIMT-1 cells treated with ZLM-7 and miR-212-3p inhibitor. Relative intensity of bands of each protein were normalized to loading control GAPDH. *P < 0.05, **P < 0.01, ***P < 0.001

We next tried to evaluate the regulatory function of ZLM-7/miR-212-3p on proliferation and angiogenesis of BC. ZLM-7 treatment decreased the proliferation of BC cells, miR-212-3p inhibitor could reduce the inhibition of ZLM-7 on the proliferation of BC cells (Fig. 2a, b). ZLM-7 treatment decreased the angiogenesis of BC cells, and miR-212-3p inhibitor could also reduce the inhibition of ZLM-7 on the angiogenesis of BC cells (Fig. 2c, d). We also detected proliferation and angiogenesis associated proteins. The expression of Sp1, VEGFA and MMP-2 in BC cells were detected by western blot and they all decreased after ZLM-7 treatment compared to control group. Compared with the ZLM-7 + inhibitor NC group, the expression of these proteins in the ZLM-7 + miR-212-3p group was significantly increased, but there was no significant difference between ZLM-7 + inhibitor NC group and ZLM-7 group (Fig. 2e, f). After ZLM-7 treatment or overexpression of miR-212-3p in tumor cells, their supernatants (also called conditioned medium) were collected and subjected to HUVECs. These conditioned mediums could inhibit the expression of VEGFR2 in HUVEC cells. Inhibiting miR-212-3p could reverse the effect of ZLM-7 (Additional file 1: Fig. S1a, b). We thus deduced that ZLM-7 inhibited the proliferation and angiogenesis of BC cells by up regulating miR-212-3p.

### MiR-212-3p reduced VEGFA expression through Sp1

**Fig. 3:**
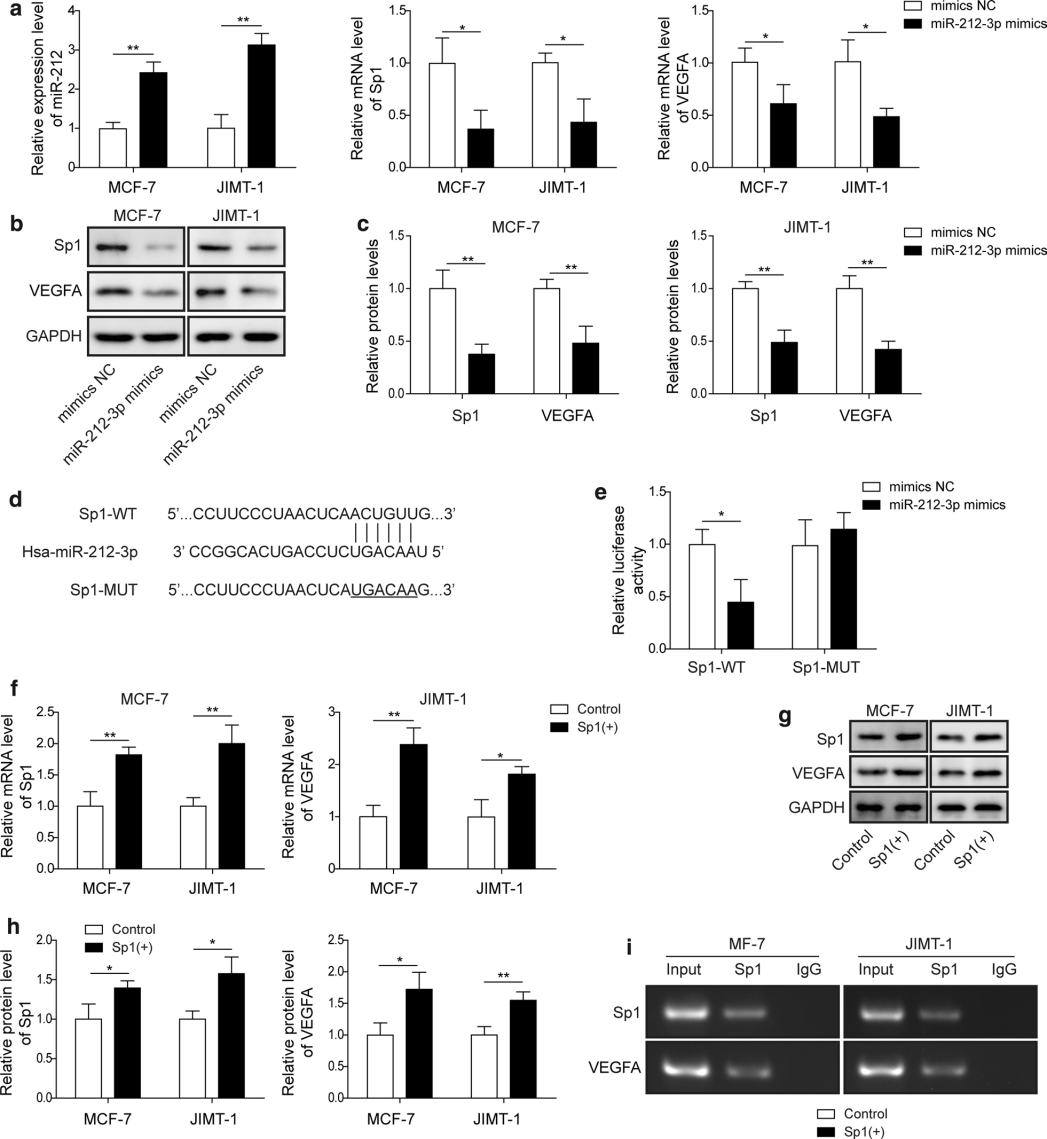
MiR-212-3p reduces VEGFA expression through SP1. **a** QRT-PCR detected expression level of miR-212-3p, Sp1 and VEGFA in MCF-7 and JIMT-1 after transfection of miR-212-3p mimics. **b**, **c** Western blot detected the protein level of Sp1 and VEGFA in MCF-7 and JIMT-1 after transfection of miR-212-3p mimics. **d** Binding site of miR-212-3p to Sp1. **e** Dual luciferase analysis of WT or MUT Sp1 in 293T cells transfected with miR-212-3p mimics. **f** QRT-PCR detected Sp1 and VEGFA mRNA levels in MCF-7 and JIMT-1 after Sp1 overexpression. **g**, **h** Western blot detected Sp1 and VEGFA protein levels in MCF-7 and JIMT-1 after Sp1 overexpression. **i** ChIP analysis of Sp1 and VEGFA promoter. Relative intensity of bands of each protein in western blot were normalized to loading control GAPDH. *P < 0.05, **P < 0.01, ***P < 0.001

Then we aimed to reveal the regulating mechanism of miR-212-3p. We speculated that miR-212-3p could inhibit expression of VEGF via targeting Sp1. In both MCF7 and JIMT-1 cells, transfection of miR-212-3p mimics resulted in increased expression level of miR-212-3p and decreased expression of Sp1 and VEGFA mRNA (Fig. 3a). After miR-212-3p mimics was transfected, the protein levels of Sp1 and VEGFA were also decreased compared with mimics NC group (Fig. 3b, c). We thus conveyed bioinformatics analysis and revealed the binding site between miR-212-3p and Sp1 (Fig. 3d). Dual-luciferase reporter system with wild type (WT) and mutated (MUT) Sp1 binding site was then constructed. MiR-212-3p mimics significantly reduced luciferase activity in Sp1-WT group, while Sp1-MUT group showed no significant difference (Fig. 3e). These results indicated that miR-212-3p could directly interact with mRNA of Sp1 and regulate Sp1 expression. We next pursued to reveal whether Sp1 could regulate expression of VEGFA. In the MCF-7 and JIMT-1 cell lines, Sp1 overexpression could up-regulate mRNA and protein level of VEGFA compared with control group (Fig. 3f–h). What's more, ChIP results demonstrated that Sp1 was bound to the promoter region of the VEGFA (Fig. 3i). Taken together, miR-212-3p could reduce VEGFA expression through targeting Sp1.

### MiR-212-3p could inhibit the migration and invasion of BC cells by reducing the expression of VEGFA through Sp1

**Fig. 4:**
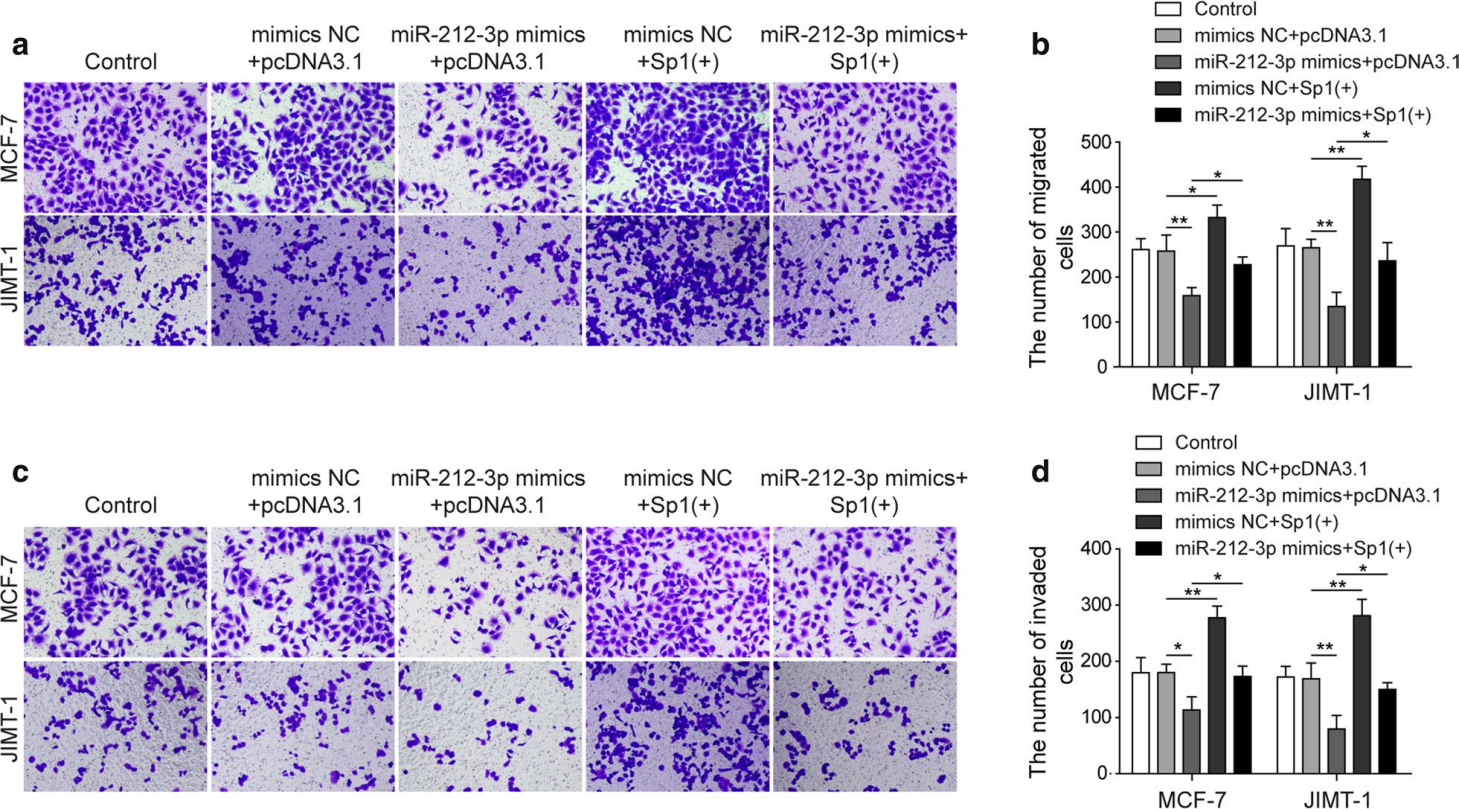
MiR-212-3p inhibited the migration and invasion of BC cells by reducing the expression of VEGFA through SP1. **a**, **b** Migration of MCF-7 and JIMT-1 was detected by transwell in cells transfected with miR-212-3p mimics and Sp1 (+). **c**, **d** Invasion of MCF-7 and JIMT-1 was detected by transwell in cells transfected with miR-212-3p mimics and Sp1 (+). *P < 0.05, **P < 0.01, ***P < 0.001

The next step, we analyzed whether migration and invasion were dysregulated by miR-212-3p. In both MCF-7 and JIMT-1 cell lines, miR-212-3p mimics successfully inhibited migration. Meanwhile, Sp1 overexpression promoted migration of these cells and antagonized the inhibitory effect of miR-212-3p (Fig. 4a, b). Similar results were observed in invasion. In both MCF-7 and JIMT-1 cell lines, miR-212-3p mimics significantly inhibited invasion, which was promoted by Sp1 overexpression. The inhibitory effect on invasion of miR-212-3p was attenuated when Sp1 was co-transfected (Fig. 4c, d). We concluded from these results that miR-212-3p could inhibit the migration and invasion of BC cells by through Sp1.

### MiR-212-3p inhibited the proliferation and angiogenesis of BC cells by reducing the expression of VEGFA through Sp1

**Fig. 5:**
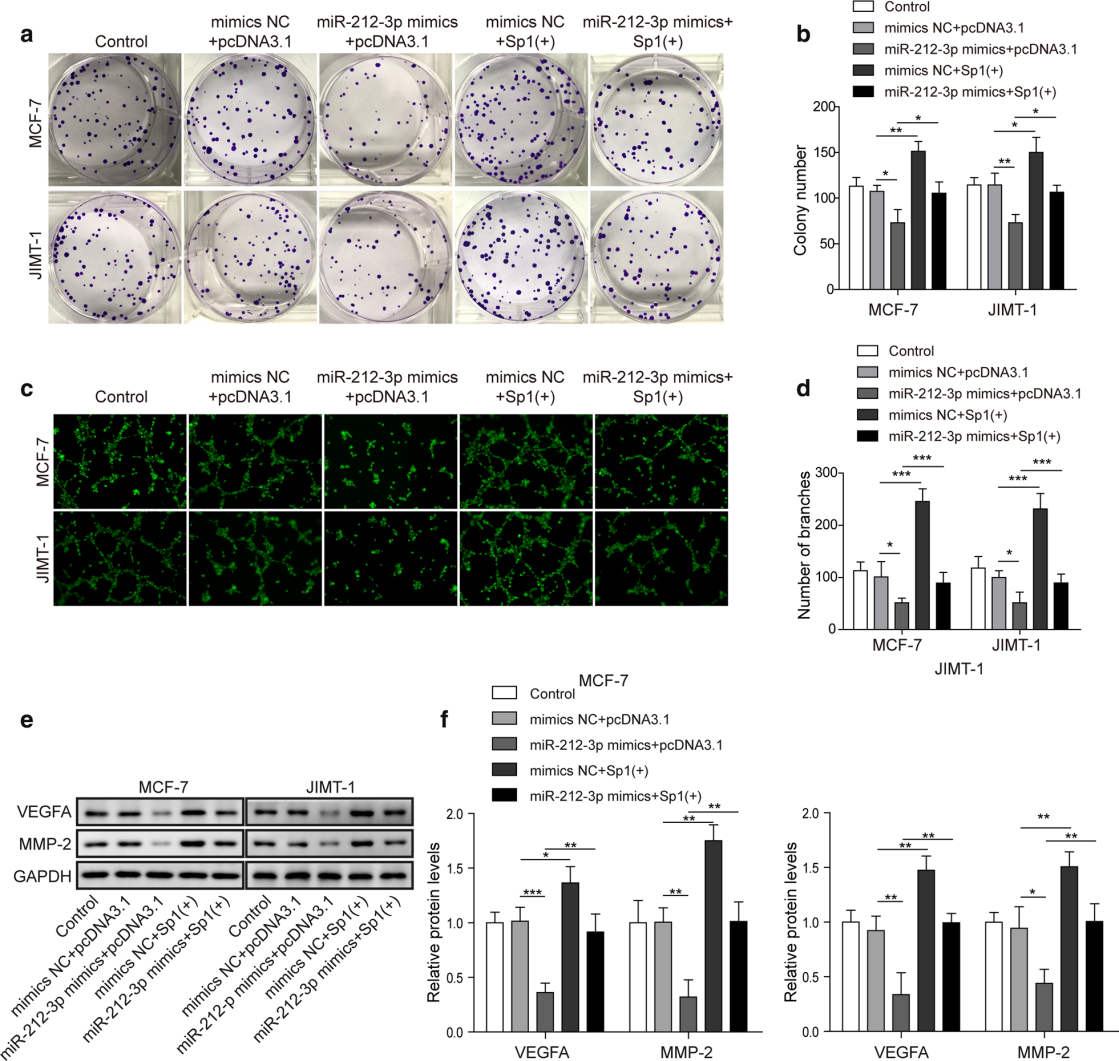
MiR-212-3p inhibited the proliferation and angiogenesis of BC cells by reducing the expression of VEGFA through SP1. **a**, **b** Proliferation ability of MCF-7 and JIMT-1 was detected by clone formation assay in cells transfected with miR-212-3p mimics and Sp1 (+). **c**, **d** Tube formation assay in MCF-7 and JIMT-1 cells transfected with miR-212-3p mimics and Sp1 (+). **e**, **f** Western blot detected VEGFA and MMP2 protein levels in MCF-7 and JIMT-1 cells transfected with miR-212-3p mimics and Sp1 (+). Relative intensity of bands of each protein were normalized to loading control GAPDH. *P < 0.05, **P < 0.01, ***P < 0.001

Besides invasion and migration, we also detected proliferation and angiogenesis of these cells. The proliferation ability of both MCF-7 and JIMT-1 cells were inhibited by miR-212-3p mimics. Sp1 overexpression promoted proliferation, and could attenuate the inhibitory effect of miR-212-3p on cell proliferation (Fig. 5a, b). The angiogenesis of BC cell lines was also inhibited by miR-212-3p mimics. Such effect was antagonized by Sp1 co-transfection, and Sp1 when transfected alone could also promote angiogenesis (Fig. 5c, d). The protein levels of VEGFA and MMP-2 were all decreased by miR-212-3p mimics and enhanced by ectopic Sp1 compared to control or mimics NC + pcDNA3.1 groups. However, when miR-212-3p mimics and Sp1 were co-transfected, expression of these proteins were rescued by Sp1 given they were higher than miR-212-3p mimics + pcDNA3.1 group (Fig. 5e, f). In addition, after overexpression of Sp1 or overexpression of miR-212-3p in tumor cells, their supernatants (also called conditioned medium) were collected and subjected to HUVECs. We found that miR-212-3p overexpression could inhibit the expression of VEGFR2 in HUVEC while Sp1 overexpression promoted VEGFR2 expression in HUVEC, besides, Sp1 overexpression antagonized the effect of miR-212-3p overexpression on HUVEC (Additional file 1: Fig. S1c, d). Thus, miR-212-3p could inhibit the proliferation and angiogenesis of BC cells by reducing the expression of VEGFA through Sp1.

### ZLM-7 reduced VEGFA expression through miR-212-3p/Sp1 and inhibited tumor growth and angiogenesis

**Fig. 6:**
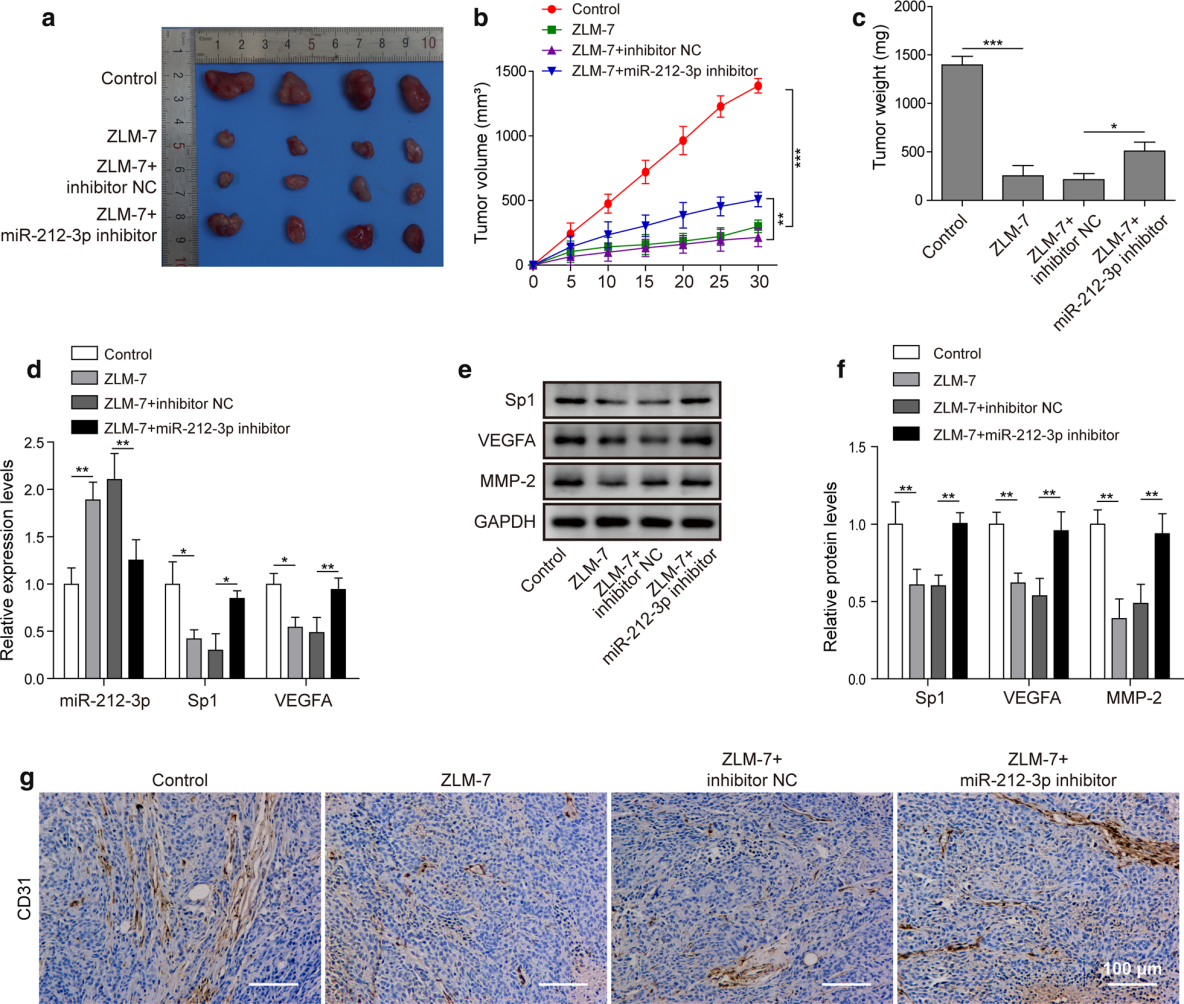
ZLM-7 reduced VEGFA expression through miR-212-3p/SP1 and inhibited tumor growth and angiogenesis. **a** Picture of tumors in mice model. **b** Tumor growth curve in indicated groups. **c** Average body weight of mice tumors in indicated groups. **d** The expression of miR-212-3p, Sp1 and VEGFA in tumor tissues of mice treated with ZLM-7 and miR-212-3p inhibitor was detected by qRT-PCR. **e**, **f** Western blot detected Sp1, VEGFA and MMP2 protein levels of tumor tissues in indicated groups. Relative intensity of bands of each protein were normalized to loading control GAPDH. **g** IHC analysis of tumor tissues in indicated groups. *P < 0.05, **P < 0.01, ***P < 0.001

To confirm whether ZLM-7 functions through miR-212-3p and Sp1, we conveyed the following experiments. In tumorigenesis experiments, tumor growth rate, volume and weight all decreased in ZLM-7 treatment group, miR-212-3p inhibitor could reduce this effect (Fig. 6a–c). The expression of miR-212-3p increased in ZLM-7 group, while expression of Sp1 and VEGFA decreased (Fig. 6d). After ZLM-7 treatment, protein expression of Sp1, VEGFA and MMP-2 in tumor tissues decreased when compared to control group. MiR-212-3p inhibitor could reduce the inhibition of ZLM-7 on the expression these proteins in tumor tissues, given expression of these proteins were higher in ZLM-7 + miR-212-3p inhibitor group than ZLM-7 + inhibitor NC group (Fig. 6e, f). In addition, the expression of CD31, an endothelial marker for quantifying angiogenesis, decreased in ZLM-7 treatment tumor tissues. MiR-212-3p inhibitor could attenuate the inhibition of ZLM-7 on CD31 expression in tumor tissues (Fig. 6g). To conclude, ZLM-7 could reduce VEGFA expression through miR-212-3p/Sp1 and inhibited growth and angiogenesis of BC in vivo.

## Discussion

BC ranks first in cancer related death in females, and is most frequently diagnosed cancer in the world (Forouzanfar et al. 2011). The prognosis of triple negative cancer was worst among all types of BC, with an overall survival of less than 18 months (Brok et al. 2017). In this study, we revealed that ZLM-7 could inhibit tumor growth and angiogenesis of BC, which largely depended on inhibitory of VEGFA expression via suppressing miR-212-3p/Sp1 signal axis.

Although publication on ZLM-7 was limited, there were publications about other microtubule inhibitors which could inhibit angiogenesis, such as SP-6-27 and MT189 (which also functions through VEGF signal) (Kulshrestha et al. 2017; Xu et al. 2018), however, their regulating mechanism was never addressed. The anti-angiogenetic effect of ZLM-7 was previously validated by chicken chorioallantoic membrane (CAM) model and MCF-7 xenograft model, presumably through inhibiting VEGF/VEGFR2 pathway (Su et al. 2016). Our findings here were much in consistence with this study. What’s more, we demonstrated that ZLM-7 could exert the ant-angiogenic effect in two independent BC cells lines, together with inhibition of proliferation, migration and invasion. At the same time, ZLM-7 could also inhibit mice tumor growth and angiogenesis in vivo. The missing link between ZLM-7 and VEGF was miR-212-3p/Sp1 signal axis. Our study firstly revealed a regulating signal axis about how microtubule inhibitors regulated VEGF signal, which was a major breakthrough, providing theological basis for future studies about microtube inhibitors and their regulation mechanism.

Meanwhile, although miR-212-3p was reported to be associated with various types of cancers such as bladder cancer, glioma, sarcoma and pancreatic cancers (Wu et al. 2019; Liu et al. 2015; Ding et al. 2018; Xie et al. 2018), its association with BC was not well understood. One weak association was that the BC resistance protein (BCRP/ABCG2) was under regulation of miR-212-3p in renal cancer cells (Reustle et al. 2018), the regulation of miR-212-3p in BC was merely mentioned. Other miRNAs also played important roles in different biological processed in BC. For instance, miR-141 was involved in migration and invasion by targeting ANP32E (Li et al. 2017b); miR-21 and miR Let-7 could serve as prognostic biomarker of BC although their mechanism was unclear (Elghoroury et al. 2018). The signal network among miRNA was extremely complexed and their functions varied a lot. Even for miR-212-3p, its function in other tissues was distinct from BC. It inhibited proliferation of glioblastoma by inhibiting SGK3, instead of inhibiting VEGF signal (Liu et al. 2015). In osteosarcoma, it promoted proliferation by targeting FOXA1 signal (Xie et al. 2018). Our finding about miR-212-3p in BC was merely revealed a small part of the whole network. In this study, we firstly validated that ZLM-7 could up-regulate the expression of miR-212-3p in BC cell lines, illustrating the regulating mechanism of miR-212-3p in BC, which we believe was a major breakthrough. In addition, inhibiting miR-212-3p could reverse the effect of ZLM-7, proving that miR-212-3p was also an important regulator of angiogenesis, proliferation, migration and invasion in BC.

The complex process of angiogenesis regulation involved multiple signaling pathways and was highly orchestrated. In this study, Sp1 was enriched in the promoter region of VEGFA and ectopic Sp1 increased the expression VEGFA, indicating that Sp1 could directly promote the expression of VEGFA. This finding was in agreement with previous reports. For instance, Sp1 promoted progression of glioma, colon cancer and ovarian cancer and in these cases Sp1 all functions through elevating VEGFA (Chen et al. 2018; Meng et al. 2019; Su et al. 2017). However, the upstream signal varied among cancers. Sp1 was targeted by miR-421 in glioma, by miR-150-5p in colon cancer, and was not demonstrated in ovarian cancer (Chen et al. 2018; Meng et al. 2019). We thereby revealed that miR-212-3p could inhibit Sp1 to inhibit BC cell lines angiogenesis, proliferation, migration and invasion, which was never reported previously. The inhibitory effect of miR-212-3p on angiogenesis was only reported in glioma but never in BC, and the mechanism was not VEGF signal dependent in this publication (Wang et al. 2019). Based on our findings, we could establish a new signal axis miR-212-3p/Sp1/VEGFA in regulation of angiogenesis of BC, which would extend our understanding of regulation function in BC.

Estrogen receptor (ER) and human epidermal growth factor receptor-2 (HER-2) are two important factors in BC (Nagini 2017; Harbeck and Gnant 2017). If ER or HER2 were positive, then the tumor growth was hormone dependent. The prognosis in ER+ patients was better than ER− patients. HER-2 was an oncogene which was usually upregulated in BC. Its expression was positively associated with tumor grade, stage and metastasis. The higher the HER-2 expression, the worse the prognosis (Harbeck and Gnant 2017). In this study MCF-7 and JIMT-1 represented ER+ and HER-2+ BC cells respectively. Selecting two types of BC cells could help us understanding whether miR-212-3p/Sp1/VEGFA signal axis could regulate different breast cancer subtypes. Results indicated that the regulatory mechanism could apply to both HER2+ and ER+ types. However, its specific mechanism and whether it has the same regulatory effect on other types of breast cancer cells may need further study in the future.

In conclusion, this study validated that ZLM-7 could inhibit angiogenesis, proliferation, invasion and metastasis of BC in both cell lines and xenograft model via inhibiting VEGFA. We also firstly proved ZLM-7 could up-regulate miR-212-3p, and miR-212-3p inhibited the proliferation and angiogenesis of BC cells by reducing the expression of VEGFA through Sp1. This discovery would enrich our understanding of the regulation mechanism of angiogenesis in BC and provided theoretical basis for future research.

## Supplementary information

**Additional file 1: Fig. S1.** Expression of VEGFR2 in HUVEC. A/B: BC cell lines were treated with ZLM-7, miR-212-3p mimics or its inhibitor as indicated. Their medium was collected as conditioned medium 24 h post transfection. HUVEC were treated with these conditioned medium. Western blot analysis in of VEGFR2 in HUVEC was then performed. C/D: BC cell lines were treated with SP1 (+), miR-212-3p mimics or its inhibitor as indicated. Their medium was collected as conditioned medium 24 h post transfection. HUVEC were treated with these conditioned medium. Western blot was performed and detected VEGFR2 protein levels in these HUVEC. Relative intensity of bands of each protein were normalized to loading control GAPDH. *P < 0.05, **P < 0.01, ***P < 0.001.

## Data Availability

All data generated or analysed during this study are included in this published article [and its supplementary information files].

## Abbreviations

BC: Breast cancer
MTA: Microtubule targeting inhibitor
CA-4: Combretastatin A-4
Sp1: Specific protein 1
VEGF: Vascular endothelial growth factor
VEGFA: Vascular endothelial cell growth factor A
WT: Wild-type
MUT: Mutant
NC: Nitrocellulose
ANOVA: Analysis of variance
CAM: Chicken chorioallantoic membrane
