# Supplementary figures and images for: ZLM-7 inhibits the occurrence and angiogenesis of breast cancer through miR-212-3p/Sp1/VEGFA signal axis
Source: Mol Med. 2020 Nov 13;26:109. doi: 10.1186/s10020-020-00239-2 (PMC7666510; doi:10.1186/s10020-020-00239-2)

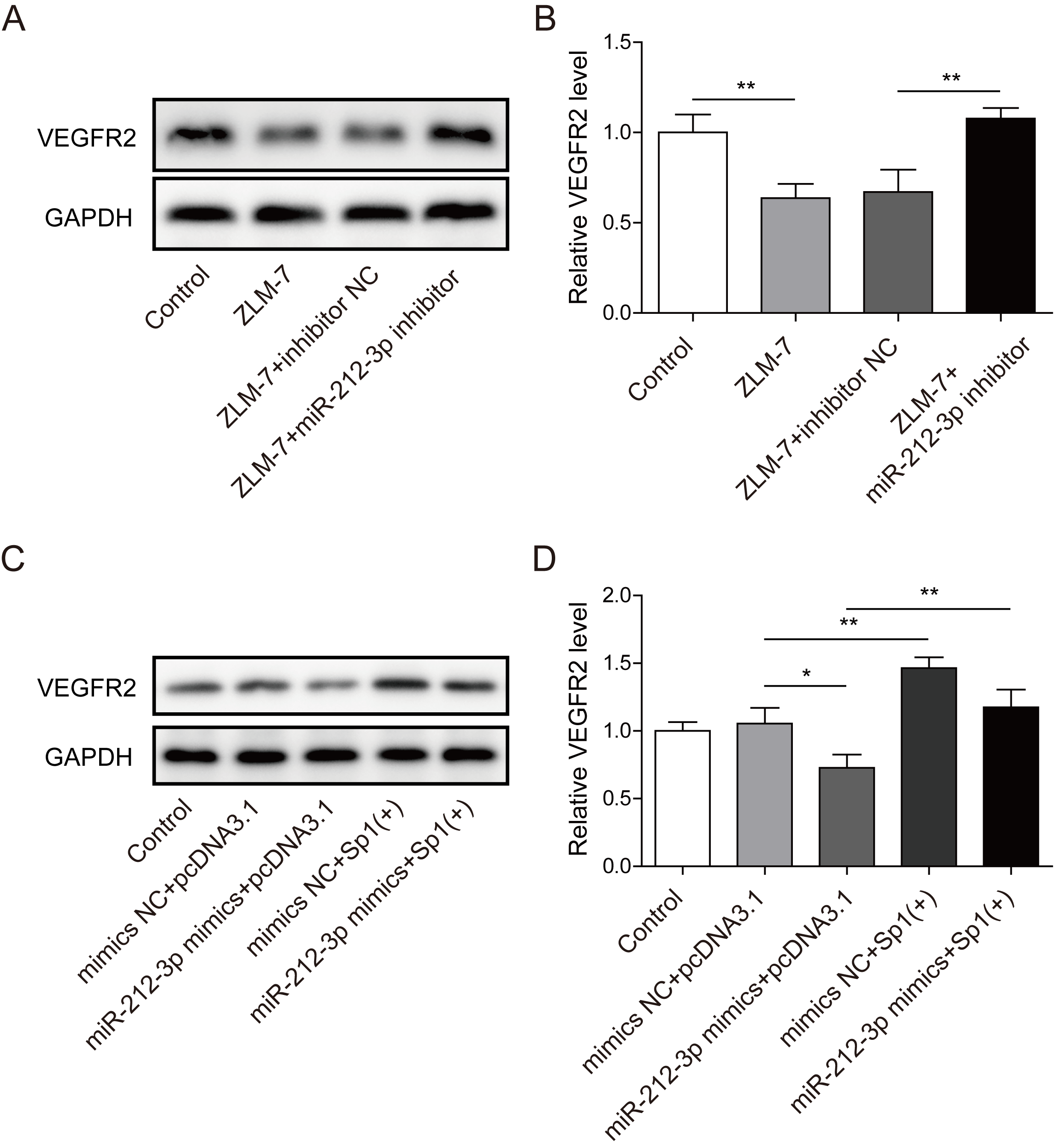

Supplement: Supplementary file 1 — Additional file 1: Fig. S1. Expression of VEGFR2 in HUVEC. A/B: BC cell lines were treated with ZLM-7, miR-212-3p mimics or its inhibitor as indicated. Their medium was collected as conditioned medium 24 h post transfection. HUVEC were treated with these conditioned medium. Western blot analysis in of VEGFR2 in HUVEC was then performed. C/D: BC cell lines were treated with SP1 (+), miR-212-3p mimics or its inhibitor as indicated. Their medium was collected as conditioned medium 24 h post transfection. HUVEC were treated with these conditioned medium. Western blot was performed and detected VEGFR2 protein levels in these HUVEC. Relative intensity of bands of each protein were normalized to loading control GAPDH. *P < 0.05, **P < 0.01, ***P < 0.001. [file 10020_2020_239_MOESM1_ESM.tif]
